# Study of knowledge, perception and attitude of adolescent girls towards STIs/HIV, safer sex and sex education: (A cross sectional survey of urban adolescent school girls in South Delhi, India)

**DOI:** 10.1186/1472-6874-8-12

**Published:** 2008-07-23

**Authors:** Alexandra McManus, Lipi Dhar

**Affiliations:** 1 Curtin Health Innovation Research Institute, Western Australian Centre for Health Promotion Research, Curtin University of Technology, Perth, Australia

## Abstract

**Background:**

Sexually Transmitted Infections (STI's), including HIV (Human Immunodeficiency Virus) mainly affects sexually active young people. Young adults aged 15–29 years, account for 32% of AIDS (Acquired Immunodeficiency Syndrome) cases reported in India and the number of young women living with HIV/AIDS is twice that of young men. The aim of the study was to evaluate adolescent school girls' knowledge, perceptions and attitudes towards STIs/HIV and safer sex practice and sex education and to explore their current sexual behaviour in India.

**Methods:**

A cross sectional study was carried out in 2007 in South Delhi, India to investigate the perception, knowledge and attitude of adolescent urban schoolgirls towards sexually transmitted Infections (STIs), HIV/AIDS, safer sex practice and sex education. the self-administered questionnaire was completed by 251 female students from two senior secondary schools.

**Results:**

More than one third of students in this study had no accurate understanding about the signs and symptoms of STIs other than HIV/AIDS. About 30% of respondents considered HIV/AIDS could be cured, 49% felt that condoms should not be available to youth, 41% were confused about whether the contraceptive pill could protect against HIV infection and 32% thought it should only be taken by married women.

**Conclusion:**

Though controversial, there is an immense need to implement gender-based sex education regarding STIs, safe sex options and contraceptives in schools in India

## Background

Sexually Transmitted Infections (STI's), including HIV (Human Immunodeficiency Virus) mainly affects sexually active young people. Young adults aged 15–29 years, account for 32% of AIDS (Acquired Immunodeficiency Syndrome) cases reported in India and the number of young women living with HIV/AIDS is twice that of young men [1]. Causes of the increased rates of STIs/HIV in young people are complex, however, the main reasons include biological factors, risky sexual behaviour patterns (early initiation of sex, premarital sex, bisexual orientation and multiple sexual partners), transmission dynamics and treatment-seeking behaviour [2]. There is growing evidence of increased premarital sexual activities among young people. While generalisation is difficult, studies indicate that between 20% and 30% of young men and up to 10% of young women have premarital sexual experiences. Women, have a higher incidence of STIs than men because of their greater biological susceptibility [3]. The present dearth of STI education in India is a major concern, particularly in schools.

Information about behaviour, attitudes, and knowledge through regular surveys is essential to better understand the dynamics of the STI epidemic. This information is also important in assessing changes over time as a result of prevention efforts. The aim of the present study was to evaluate adolescent school girls' knowledge, perceptions and attitudes towards STIs/HIV and safer sex practice and sex education and to explore their current sexual behaviour in India.

## Methods

With an increasing number of people living with HIV/AIDS, Delhi is categorised as a highly vulnerable state for the disease [4]. A cross sectional survey was carried out in January 2007 in two urban higher secondary schools in South Delhi, India. Permission to conduct the survey was granted in accordance with the requirements of the Education Department governing the schools involved. The sample comprised 251 female students from two senior secondary schools, one a co-education school (n = 99) and the other an all girls' school (n = 152). Within the two schools 41% (n = 102) of participants were from 11th grade and 59% (n = 149) were from 12th grade.

Independent, trained research assistants distributed the questionnaire and an envelope to the students and remained in the classroom during survey completion. A self administered, English language (at the secondary level all students can read and write basic English) questionnaire comprising mainly multiple-choice items was developed for the study. Most of the items were adapted from the Questionnaire for Australian Secondary Students, HIV/AIDS and Sexual Health Survey, prepared by Australian Research Center in Sex, Health and Society, La Trobe University and modified to suit Indian adolescent girls.

As the survey covered sensitive issues, and to avoid confounding through discussion amongst participants, data were collected at the same time, on the same day in each school. All students who were present in the classroom on the day of the survey were asked to complete a questionnaire. There were no refusals. At the beginning of each session, an information statement was read to students outlining the aim of the survey and the reason respondents had been selected. In order to promote candid responses, students were assured anonymity and were free to refuse to complete the questionnaire or any particular question(s). Students were instructed to place their completing questionnaire into the envelope provided and seal it. The sealed envelopes were collected by the independent researchers and transported to the School of Public Health, Curtin University of Technology, Perth, Australia.

All data were entered using SPSS (Statistical Package for the Social Sciences, Version 12) [5] and checks performed to ensure accuracy. Frequencies were assessed for each question to verify that responses were within the defined range of possible values. Incorrect entries were examined and verified against the original questionnaires. In addition all missing responses were recorded and checked against the original questionnaire.

### Ethical considerations

Ethics approval was received from the Curtin University Human Ethics Committee and the State AIDS Control Society, Delhi to conduct this research. Permission to carry out the research was also obtained from the principals of the schools involved. (Principals have the authority to allow cross sectional surveys to be conducted without parental consent in India.) This complies with the Helsinki Declaration for research conducted with humans. All participants provided written consent prior to being involved in this study and all results were de-identified prior to reporting.

## Results

The mean age of the responding students was 16.38 years (SD = 0.945) and ranged from 14 to 19 years. Most of the students (n = 176; 70%) identified themselves as Hindu. Among others 14% (n = 36) of the students were Muslim, 6% (n = 15) Christian, 5% (n = 13) Sikh, 2% (n = 6) Jain and 1% (n = 3) Buddhist. One student reported no religious affiliation and one student did not response. A summary of the main findings of the study is provided herein.

The majority of respondents (71%, n = 179) had no knowledge about the effects of Genital Herpes infections, two fifths did not know the consequences of acquiring Syphilis (43%, n = 107) and 28% (n = 71) were unaware that Gonorrhoea was an STI. One third of the girls (33%, n = 82) did not consider ulcers in the genital area and pain during urination (31%, n = 78) as sign and symptoms of STIs in women. In addition, 22% (n = 54) of the girls did not know vaginal discharge was an important sign of STIs in women. Alternatively, students in the survey reported chest pain (24%, n = 61) and throat pain (9%, n = 23) as symptoms of STIs in women.

In response to the question 'people who always use condoms are safe from all STIs', 19% (n = 47) of respondents said this was not true while 22% (n = 54) were unsure. Furthermore almost half of the girls (46%, n = 116) said that apart from HIV/AIDS, all other STIs could be cured. Only 14% (n = 36) of the respondents had the correct information about this topic. More than half of the adolescent girls (59%, n = 147) in the study did not know that some STIs can lead to sterility while 26% (n = 66) were unsure.

Although 28% (n = 71) of girls believed that it was not possible for girls to remain a virgin prior to marriage, 9% (n = 22) said they were sexually active. When asked questions regarding HIV prevention, 21% (n = 52) considered that the contraceptive pill could protect a women from HIV infection while 41% (n = 102). Eighteen percent (n = 44) and 31% (n = 77) of students in the survey did not know that condoms were an effective method of preventing HIV/AIDS and other STIs respectively.

The main sources of information available to respondents about HIV/AIDS, other STIs and safer sex were friends (76%, n = 191), the media (72%, n = 1680), books/magazines (65%, n = 165) and the Internet (52%, n = 132). Forty one percent (n = 104) of students had not used teachers as a source of information. Half of the adolescent girls (48%, n = 119) considered that it was not possible to talk with their parents about sex and STIs however 24% (n = 61) had used their mother as a source of information. Nine percent (n = 23) of students preferred lady doctors as their source of information. While one student remarked that "I am a girl so I want to receive information from an experienced lady doctor".

## Discussion

STIs (including HIV) can be controlled by reducing the risk of transmission in any sexual encounter (such as condom use); reducing the rate of sexual partner change and reducing the period of infectiousness in individuals [6]. Education is essential to assist young people to make informed decisions about their sexual health [7].

### HIV/AIDS related knowledge

The findings of this study indicated good awareness about the modes of HIV transmission and prevention among adolescent girls. For example, 77% (n = 193) of girls were aware of the link between high-risk behaviour such as multiple sex partner and HIV. Conversely, in a study published conducted by the ICMR (Indian Council of Medical Research) in higher secondary schools in rural areas of 22 districts and 14 states, only 13% of adolescent knew that multiple sex partners increased the risk of HIV infection [8].

Some areas still needed special attention. Approximately half of the adolescents (46%, n = 111) in this study were not sure if a healthy looking HIV infected person could transmit the HIV infection to others. In a study among school children in the state of Hariana, 57% believed that persons with HIV/AIDS could be detected by their physical appearance [9]. This is of concern as evidence shows that people with HIV may remain asymptomatic for several months or years before developing AIDS but still transmit the infection[7]. A significant number of respondents in this study (n = 30%, n = 75) also considered AIDS could be cured. Other surveys within India show similar [9]. This is clearly an area that requires attention.

There was also a lack of knowledge about the risk of transmission of infection among MSM (men who have sex with men). In 2002, a study found that the HIV prevalence among MSM in the City of Mumbai was 20% [10]. Research shows MSM were eight times more likely to be seropositive for HIV and over twice more likely to have a history of STIs compare with non – MSM [11].

It is important to note that among the school course elements that have generated most controversy and debate in India are discussions about homosexuality and information on the options of safer sex, including condom use and masturbation.

### Sexual behaviour

Research indicated that attitudes, outlooks, norms and beliefs around sexual behaviour determines the intended sexual behaviour of young people [12]. In this study 15% (n = 37) of respondents did not believe that girls should remain a virgin until they marry while 9% of girls admitted a sexual experience. However, several studies have indicated that the most common reasons cited for having engaged in unwanted sex were being drunk and pressure from a sexual partner [13].

Bridging the gap between knowledge and practice (particularly with respect to the use of condoms) has emerged as a major behavior change communication challenge to reducing adolescents' vulnerability to STIs and unwanted pregnancies [14]. The present survey showed 8% (n = 20) of students felt that no one (of their age) used condoms if they had sex while 33% (n = 82) considered that a few students used condoms during sex.

### Knowledge about STIs other than HIV/AIDS

The findings of this study indicated that knowledge about STIs other than HIV/AIDS was very poor among adolescent girls. The majority (71%) had not heard about Genital Herpes and almost half had not heard about Gonorrhoea (44%) or Syphilis (43%). This is of particular concern in developing countries like India, as STIs such as Chlamydia, Trichomoniasis, Syphilis and Gonorrhea are second only to maternal morbidity and mortality as the cause of death, illness and 'years of healthy life lost' among women in their child bearing years [15].

Comparison of findings is difficult as there are no published studies in India investigating the STI knowledge (other than HIV/AIDs) of adolescent schoolgirls. More than one third of student in the present study had no accurate understanding about the signs and symptoms of STIs. The only study with some comparability was conducted by Lal et. al., (2000) in Kerala where college students had 34% awareness of the symptoms of STIs [16].

The present study found that a number of students (22%, n = 54) did not know that people who always used condoms were safe from all STIs. However, male and female condoms are the only technology available that can prevent sexual transmission of HIV and other STIs [17]. Laboratory studies have found that STIs do not pass through intact latex condoms even when devices are stretched or stressed [17]. They reduce the risk of transmission of HIV, Gonorrhea and Chlamydia [18] and Herpes simplex virus [19] in both women and men during sexual intercourse. Young sexually active people need to be aware of the risk of STIs and correct use of condoms to prevent STIs transmission. The continued promotion and widespread availability of condoms is an essential STIs control strategy [20].

### Social norm

Attitudes, norms and motivational factors are crucial elements in the decision making process of adolescents around engaging in risky behaviours [21]. Interestingly, in the present survey, almost one quarter of the adolescent respondents (22%, n = 55) had agreed that there was nothing wrong with unmarried boys and girls having a sexual relationship if they loved each other. This observation is reflected in the increasing incidence of premarital sex in India.

When asked if students should have access to condoms, surprisingly, 49% (n = 123) of respondents felt that condom should not be available to youth because it encouraged them to have sex. In a similar study conducted in both India and Kenya, there was also a strong resistance towards the availability of condoms in both countries [22].

Though contemporary literature reveals that oral contraceptives are safe for adolescents, in India, less than 10% of adolescents use any form of contraceptive [23]. In the current study 32% (n = 82) of girls thought that girls should not take the contraceptive pill as it should only be taken by married women. Adolescents' attitude towards contraceptives (including condoms) may be based on cultural and other beliefs that need specific educational efforts to change.

### Sources of information

The result of the present study indicated that for adolescent students the Internet, media, friends, books and magazines were the main sources of information regarding safe sex and HIV/STIs. Often students were confused or misinformed due to erroneous information received from these sources. Therefore evidence-based sex education must be a major strategy in school-based programs, with user friendly resources ready available to students.

Currently HIV/AIDS education is only taught in science which is an optional subject in India. In the current study more than half of the adolescents (53%, n = 132) had never attended classes about STIs, HIV/AIDs or safe sex. Most of the adolescent (87% n = 215) felt that there should be classes related to HIV/AIDS, other STIs and safe sex in school. This findings is supported by a study conducted by the Kore et. al., (2002) where 71% students showed a willingness to attend awareness programs arranged related to HIV/AIDS [24].

In order to intensify the focus on STI prevention among young people, in June 2005, the Government of India announced the National Adolescent Education Program (AEP). While the main focus of the program was on HIV/AIDS prevention, it also covered sexual reproductive health issues, gender and life skills [10]. Regrettably the proposal to incorporate compulsory sex education in the school curricula as part of anti-AIDS course was rejected outright by State Governments of Madhya Pradesh [25], Maharashtra [26] and Gujarat [27,28] even before implementation of the program. Kerala and Karnataka [27], India's progressive states, were also considering sex education bans. [29]

The opponents of sex education in schools say that the AEP is not culturally sensitive, it has ambiguous material and could encourage students to experiment with sex, defeating the very purpose of the campaign and contributing to the spread of HIV/AIDS. Reacting to the Central Government's circular for immediate implementation of the AEP in all CBSE (Central Board of Secondary Education) and state-syllabus schools, Karnataka Education Minister Basavaraj Horatti said, "In today's world, we need moral education and not sex education" [27]. He also claimed that school-going children were too young to contract the disease, therefore sex education would unnecessarily affect their tender minds [27]. Most Indian states are yet to make a decision about introducing sex education [26]. If schools do agree to implement the AEP, further training in communication skills for educators/teachers and health personnel will be required. In addition, support of state political leaders and health and education authorities is essential for STI prevention interventions to be effectively executed in Indian schools[30].

Unfortunately, many school students are exposed to sex and pornography through various television channels, cell phones and Internet cafes. Educating adults and children is essential under these non-regulated conditions. According to AIDS activists, sex education helps to make students cautious against the dangers of experimenting with sex at a young age, sensitising them and also warning them about the potential exposure to deadly diseases. Many parents are hesitant to talk about sex with their teenage children at home; even mothers hesitate to talk to daughters about something as simple as menstruation. Moreover, widespread illiteracy underlines the importance of being able to talk about sexuality comfortably in a gathering or congregation. Policymakers also believe that many people will be critical of moves to implement sex education to young people therefore may seek political advantage by promoting traditional values.

## Conclusion and recommendations

In spite of efforts over the past two decades in STI prevention, this epidemic still presents a serious challenge to societies around the world, including India. Every year, increasing numbers of young people are infected with HIV and other STIs. Global surveillance and research has identified adolescents (particularly girls) as an emerging high-risk or vulnerable group. Sex education and STI education aimed at adolescents is a crucial weapon in the STIs/HIV-prevention armoury and the school is an important means of reaching them. A number of studies, including the current study identified lacunae in the STI/HIV knowledge and reflected low levels of awareness among adolescent girls. It is important to educate adolescents about safe sex and contraceptives so that they can safeguard themselves from STIs by practicing safe sex or monogamy. It is also essential to provide information about signs and symptoms of STIs which will alert them to seek timely medical attention as needed. The present strategy of STIs education in India is simply not effective.

In summary, it may be said that despite all opposition there is an immense need for implementation of appropriate gender-based, culturally sensitive sex education curriculum in schools to cope up with the increasing vulnerability of young adults, especially girls, towards STIs/HIV in India. Standardised programs across all levels of secondary schools in India will allow young people to make informed choices about protecting themselves from STIs/HIV if or when they decide to become sexually active.

## Competing interests

The authors declare that they have no completing interests.

## Authors' contributions

AM supervised the research project and assisted via expertise in survey instrument development and data analysis. LD conducted the study as part of a Master of Public Health degree. All authors read and approved the final manuscript.

## Pre-publication history

The pre-publication history for this paper can be accessed here:


